# A Single Dose of Modified Vaccinia Ankara Expressing Lassa Virus-like Particles Protects Mice from Lethal Intra-cerebral Virus Challenge

**DOI:** 10.3390/pathogens8030133

**Published:** 2019-08-28

**Authors:** Maria S. Salvato, Arban Domi, Camila Guzmán-Cardozo, Sandra Medina-Moreno, Juan Carlos Zapata, Haoting Hsu, Nathanael McCurley, Rahul Basu, Mary Hauser, Michael Hellerstein, Farshad Guirakhoo

**Affiliations:** 1 Institute of Human Virology, University of Maryland, Baltimore, MD 21201, USA; 2 GeoVax, Inc., Smyrna, GA 30080, USA; 3 Office of Technology Licensing and Commercialization, Georgia State University, Atlanta, GA 30303, USA; 4 Department of Biology, Georgia State University, Atlanta, GA 30302, USA

**Keywords:** Lassa vaccine, replication-deficient MVA vector, VLP formation, single-dose efficacy, cell-mediated immunity

## Abstract

Lassa fever surpasses Ebola, Marburg, and all other hemorrhagic fevers except Dengue in its public health impact. Caused by Lassa virus (LASV), the disease is a scourge on populations in endemic areas of West Africa, where reported incidence is higher. Here, we report construction, characterization, and preclinical efficacy of a novel recombinant vaccine candidate GEO-LM01. Constructed in the Modified Vaccinia Ankara (MVA) vector, GEO-LM01 expresses the glycoprotein precursor (GPC) and zinc-binding matrix protein (Z) from the prototype Josiah strain lineage IV. When expressed together, GP and Z form Virus-Like Particles (VLPs) in cell culture. Immunogenicity and efficacy of GEO-LM01 was tested in a mouse challenge model. A single intramuscular dose of GEO-LM01 protected 100% of CBA/J mice challenged with a lethal dose of ML29, a Mopeia/Lassa reassortant virus, delivered directly into the brain. In contrast, all control animals died within one week. The vaccine induced low levels of antibodies but Lassa-specific CD4^+^ and CD8^+^ T cell responses. This is the first report showing that a single dose of a replication-deficient MVA vector can confer full protection against a lethal challenge with ML29 virus.

## 1. Introduction 

Lassa fever (LF), a zoonotic disease caused by Lassa virus (LASV), can lead to acute hemorrhagic fever with a case fatality rate (CFR) of up to 50% [1]. The estimated annual incidence of LF across West African countries including Ghana, Guinea, Mali, Benin, Liberia, Sierra Leone, Togo, and Nigeria has been reported as high as 300,000 infections and 5000–10,000 deaths, but these figures are most likely underestimates due to inadequate diagnosis and surveillance [2,3,4,5]. Based on prospective studies performed in Guinea, Sierra Leone, Liberia, and Nigeria, it was estimated that 59 million people are at risk of LASV infections, with as many as 67,000 deaths per year [6]. The 2018 outbreak in Nigeria resulted in 3498 suspected cases in 22 states and 171 deaths with a CFR of 27% based on confirmed cases [7]. The fact that a virus that was first discovered in 1969 in Lassa village in Nigeria is still capable of causing outbreaks in the same geographical regions indicates challenges in eradicating its animal reservoir. 

The main animal reservoir of LASV is the multimammate rat (*Mastomys natalensis*), which is common in West Africa, but the potential transmission of LASV to new rodent hosts could have serious implications for its spread beyond West Africa [8]. LASV is mainly transmitted to humans by consumption of contaminated food, by contact with rodent urine and droppings, or by inhalation of virus particles from the excreta of infected animals; it can also be transmitted from human to human through nosocomial infections [9]. Most LASV infections are asymptomatic or cause only mild symptoms, but some infections lead to multi-organ failure, fever, and, on occasion, hemorrhage and death [10,11].

LASV is a member of the family *Arenaviridae* and genus *Mammarenavirus,* which includes Old World and New World arenaviruses [12,13]. Furthermore, LASV strains are classified into four lineages, I–IV (with a newly proposed fifth lineage from Cote d’lvoire and Mali) based on their genetic variations [12,13]. The LASV genome is composed of two ambisense RNA segments. The large (L) segment of 7.2 kb in length encodes the viral RNA-dependent RNA polymerase protein and the zinc finger (Z) protein, a multifunctional matrix protein [14,15]. The small (S) segment of 3.4 kb in length encodes the nucleoprotein (NP) and glycoprotein precursor (GPC). GPC is cleaved post-translationally to form a trimer of hetero-trimers, each consisting of a receptor binding protein (GP1), a fusion protein (GP2) and a stable signal peptide (SSP) [16,17]. GPC sequences, shown previously to be required for full protection, have been used as the protective antigen in the construction of a large number of vaccine candidates delivered by various platforms. Some of these candidates have undergone preclinical evaluations in mice, guinea pigs, and non-human primates (NHP) [12], and a DNA vaccine has entered clinical trials [18]. 

Here, we describe the construction, characterization and efficacy of a Lassa vaccine candidate that generates virus-like particles (VLPs) by expression of Lassa GPC and Z proteins from our Modified Vaccinia Ankara (MVA) vector. It has been previously demonstrated that co-expression of Z and GP proteins leads to the formation of VLPs [19]. A murine model was chosen initially to establish dose and route of vaccination and whether our constructs could elicit any protective immunity. In this model, ML29, an attenuated Mopeia -Lassa (MOP/LAS) reassortant, is delivered into the brain and primarily infects brain capillary endothelial and glial cells that present LASV antigens, triggering an influx of lymphocytes 5–6 days later. The infiltrating cells contribute to encephalitis that is lethal unless the mice had been previously vaccinated against LASV (Djavani and Salvato, unpublished). The intra-cerebral (IC) delivery we used was not “natural”, since it models brain infections that are rare in LF, but it allows a rapid and simple read-out for whether or not we elicited a protective immunity. Here, we show that a single dose of the vaccine conferred full protection against a lethal challenge dose of the ML29 virus delivered IC. 

## 2. Results

### 2.1. Vaccine Characterization

**Figure 1:**
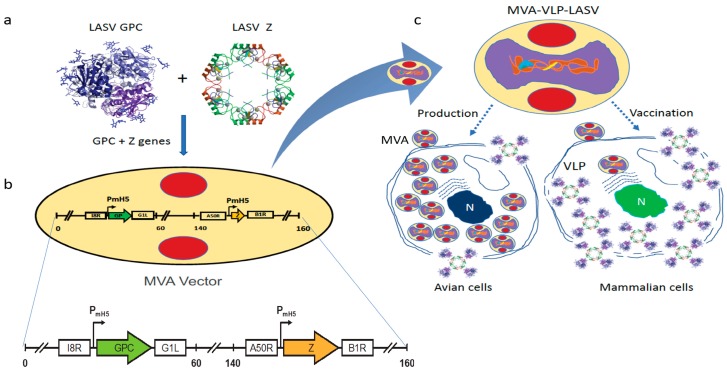
**Construction of GEO-LM01 vaccine candidate**. A Cartoon showing the vector design. (**a**,**b**) The Lassa virus (LASV) sequence for glycoprotein precursor (GPC) was inserted between the I8R and G1L genes and the Z sequence was inserted between the A50R and B1R genes. P_mH5_, modified H5 promoter. Numbers are coordinates in the Modified Vaccinia Ankara (MVA) genome. (**c**) Similar to all MVA-derived vaccine viruses, GEO-LM01 is replication-competent in avian cells, used for propagation of the vaccine but replication-deficient in mammalian cells that are used for vaccination purposes. Image of the LASV GPC protein structure is courtesy of Dr. Erica O. Saphire, The Scripps Research Institute. The image of LASV Z structure is from RCSB PDB (www.rcsb.org) ID 5I72 [22].

**Figure 2:**
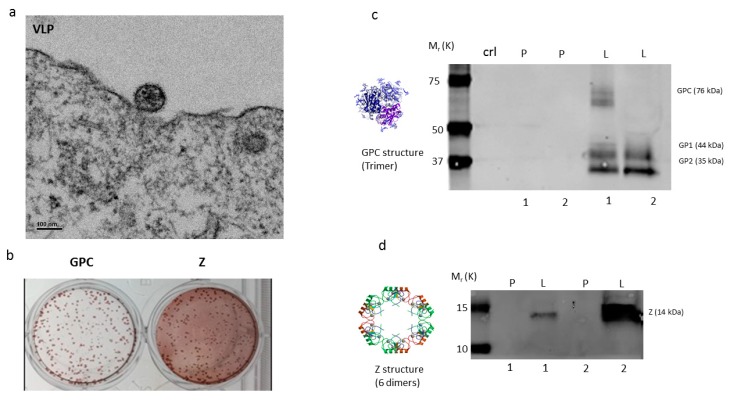
**Electron microscopy, Immunocytochemistry and Western blot of GEO-LM01 vaccine candidate**. (**a**) Electron micrograph of virus-like particles (VLP) formed in cells infected with GEO-LM01. (**b**) Immunocytochemistry on infected duplicates of cell monolayers stained with GPC- (left) or Z- (right) specific antibodies. Western blots verified expression of LASV GP (**c**) and Z (**d**) proteins in cultured cells. DF1 cell lysates contained both the unprocessed GPC and the processed subunits GP1 and GP2, whereas the DF1 supernatants contained only the processed GP1 and GP2 subunits. P, Parental (empty) MVA; L, GEO-LM01; 1, cell lysate; 2, supernatant. A loading control lane (**crl lane c**) served as another negative control. The two GP bands correspond to LASV GP1 and GP2 with the molecular weights of 44kD and 35kD, respectively.

The recombinant GEO-LM01 was constructed by inserting sequences from the well-characterized LASV prototype strain Josiah from lineage IV into MVA. The Josiah strain is extensively used for vaccine development, since it has shown protection against homologous and heterologous challenges [20]. The GPC protein of Josiah, the major protective antigen, has 93.1%, 93.5, and 94.5% homologies with lineage I, II, and III, respectively [12]. GEO-LM01 is designed to co-express GPC and the LASV matrix protein Z (Figure 1a,b). Expression of GPC and Z lead to the formation of VLPs in the cells of the inoculated host as well as promoting their release from GEO-LM01-infected cells. GEO-LM01 is replication-competent in avian cells (producing both infectious progeny and expressed antigens) but is replication-deficient in mammalian cells (producing antigens in the form of VLPs but not infectious progeny) (Figure 1c) due to six deletions in the MVA genome which together resulted in high attenuation and mammalian cell host restriction [21]. The assembly of VLPs from proteins expressed by GEO-LM01 was verified by electron microscopy, demonstrating active release of VLPs from DF1 cells, a chicken fibroblast cell line (Figure 2a). The entire population of GEO-LM01 virions retains both GPC and Z transgenes as demonstrated by immunostaining (Figure 2b). Similar plaque numbers at each dilution in the GPC and Z wells showed that both transgenes are equally retained. Expression of both transgenes and cleavage of GPC into GP1 and GP2 subunits was further demonstrated in western blots (WB) using cell lysates and supernatants (released VLPs) of GEO-LM01 infected DF1 cells (Figure 2c,d). 

### 2.2. Vaccine Efficacy Testing in a ML29 Mouse Challenge Model

**Figure 3:**
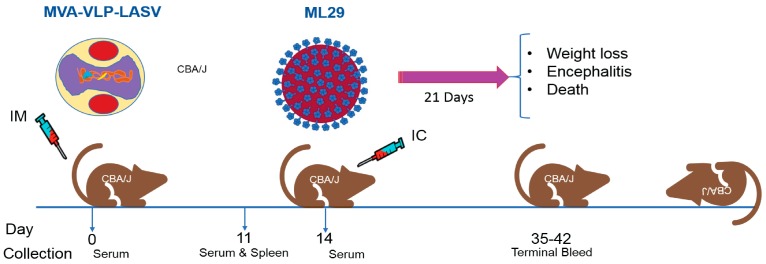
**ML29 challenge model; schedule of vaccination, sample collections and intracerebral (IC) challenge.** CBA/J mice are vaccinated with a single dose of GEO-LM01 (MVA-VLP-LASV) (1 × 10^7^ TCID_50_ in 100 µL volume) by the intramuscular (IM) route. Three mice are sacrificed on day 11, sera and spleens were collected for assessment of antibody and T cell responses. The remaining mice are challenged on day 14 by the IC route with 1000 plaque forming units (PFU) of ML29 virus. After challenge, mice are observed for weight loss, signs of encephalitis and death.

Immunogenicity and efficacy testing of GEO-LM01 was performed in a mouse model that uses ML29 virus for lethal challenge (Figure 3). In this model, young immune-competent CBA/J mice are vaccinated once and challenged 2 weeks later. Animals are monitored for weight loss, morbidity, and mortality. Blood and spleens from three mice were collected on day 11 prior to challenge for determination of vaccine-elicited antibody and T cell responses, respectively.

**Figure 4:**
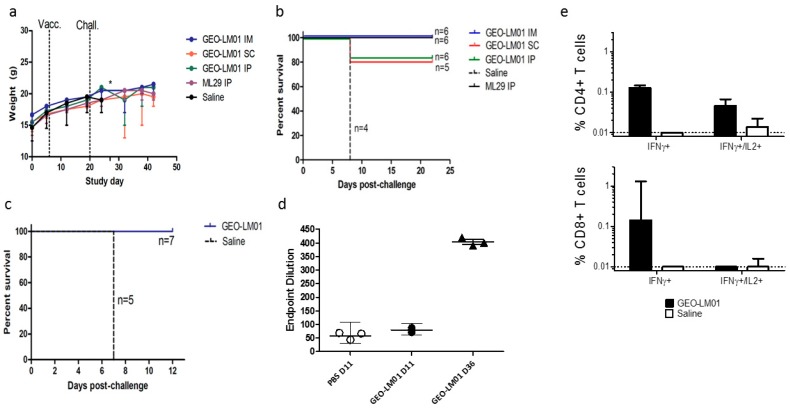
**Efficacy and immunogenicity of GEO-LM01.** Weight loss (**a**) and survival curves (**b**) following immunization by various routes then challenged with IC with ML29 virus 14 days later. * demonstrates death of control animals. (**c**) Survival curves of animals vaccinated by IM route only. (**d**) ML29-specific Ab titers from immunized on days 11 and 36 (22 days after challenge) and mock-immunized mice on day 11 as determined by ELISA. (**e**) Percentage of antigen-specific CD4+ and CD8+ T cells expressing IFNγ and IL2 in response to LASV GP peptides from GEO-LM01 or saline immunized animals were assessed by flow cytometry. These data demonstrate considerable differences between immunization groups but are not statistically significant.

ML29 is a reassortant virus encoding the GPC and NP proteins of LASV (Josiah strain) and the L and Z proteins of MOPV virus [23]. The virus is uniformly lethal when administered by IC inoculation into immunocompetent CBA/J mice; however, when administered by intraperitoneal (IP) inoculation, ML29 elicits a strong immune response that protects CBA/J mice from death upon subsequent IC challenge [24]. To determine the best route of immunization, 4–6 week-old CBA/J mice were immunized with 10^7^ TCID_50_ of GEO-LM01 by IP, IM, or SC inoculation. Additionally, two groups of mice (n = 6) were injected IP with ML29 virus (1000 PFU) or saline, and served as positive and negative controls, respectively. On day 14, all mice were challenged by IC inoculation with ML29 virus and monitored for weight change, morbidity, and mortality. Mice immunized with ML29 virus (IP) (positive control) or with GEO-LM01 (IM) were 100% protected from lethal challenge and gained weight throughout the study (Figure 4a), whereas mice administered saline alone all died on day 7 or 8 after lethal challenge (Figure 4b). Some mice in the vaccinated groups exhibited minor weight loss after challenge, but they all regained weight and remained healthy (no ataxia or ruffled fur) until the end of the study. Mice immunized with GEO-LM01 by SC and IP administration showed less robust immunity to lethal challenge, as seen in the more appreciable weight loss in these groups as well as the death of one animal in each group (one animal in the SC group died early on day 2 post challenge, most likely due to problems with inoculation rather than virus growth; it was excluded from survival analysis). A second experiment to measure immunogenicity and efficacy was initiated in the same model system, in this instance examining only two conditions: Immunization with GEO-LM01 by IM inoculation (n = 10) and mock immunization with saline (n = 8) (Figure 4c). Spleens were harvested from immunized animals (n = 3) and saline-inoculated animals (n = 3) on day 11 after a single administration of the vaccine. 

Antigen-specific T cell responses were measured by Intracellular Cytokine Staining (ICS) for IFNγ and IL2 production by stimulating with peptides derived from immuno-dominant epitopes (IDE) present in GP1 and GP2 subunits [24,25,26]. IFNγ expression was evident in both CD4+ and CD8+ T cells of immunized but not mock immunized mice (Figure 4e). Some CD4+ T cells were also shown to be double positive for IFNγ and IL2, suggesting an expanding population. The remaining seven vaccinated and five control animals were challenged on day 14 as in the first experiment. All vaccinated animals survived the challenge, whereas all controls died by day 7 (Figure 4c) confirming the 100% single-dose efficacy of GEO-LM01 observed in the previous study. Four of the surviving GEO-LM01-vaccinated mice were housed for an additional year to see if they would be able to survive a second challenge long after the initial challenge. All four survived their second IC challenge (Table 1). A true test of durable immunity would not use mice surviving lethal challenge because the ML29 challenge itself may boost vaccine immunity; a better test would be to vaccinate mice and leave them unchallenged for a year to see whether or not the single vaccination could confer durable year-long protection.

We additionally assayed for the presence of binding antibody (bAb) to GPC in the serum of the immunized mice. Despite the excellent protection from death afforded by GEO-LM01, we found no statistically significant level of bAb above background, we found low bAb at the early sampling days 11–14 post vaccination (Figure 4d). Similar results were observed previously with an ML29 vaccine that conferred protection from lethal challenge but yielded a minimal amount of bAb and neutralizing antibody (nAb) [24]. Substantial bAb were found in mice 36d after vaccination (Figure 4d) but these had been ML29 challenged, which could serve to boost the response to LASV antigens. We did not look for neutralizing antibodies.

## 3. Discussion

LF has a greater human impact than any other hemorrhagic fever, with the exception of Dengue fever [6]. Despite the severe impact of LF on populations in West Africa, no effective and practical medical countermeasure is available [18]. The one available treatment is the antiviral drug ribavirin, which is effective only if given within six days after infection [18]. A vaccine against LASV would be an ideal solution to the problem of LF, especially given the growing threat of outbreaks due to globalization and increased travel [27]. Several groups have developed vaccine candidates that have shown activity against LASV in animal models [20,23], but only one has progressed to the clinic [18]. Although a multi-dose immunization regiment is logistically feasible for medical providers and military personnel, a single dose vaccine is critical for epidemic response and is preferable and much more practical in under-resourced endemic areas. LF has been listed in the WHO R&D Blueprint among the 10 diseases and pathogens to prioritize for research and development in public health emergency contexts [28]. These diseases are identified as those that pose a public health risk because of their epidemic potential and for which there are insufficient countermeasures. For this reason, CEPI (Coalition for Epidemic Preparedness Innovations), founded to address the threat of epidemic diseases, has set aside a major portion of its $1 billion budget for the development of vaccines against LF [29]. 

GEO-LM01 provides a unique combination of advantages required for visitors or residents of LASV endemic countries. The potential for GEO-LM01 to protect after a single dose is favorable for travelers and military personnel who require a rapid onset of immunity (ideally ~<2 weeks post vaccination) as well as for epidemic/endemic vaccinations (due to the urgency of epidemic response and the complexity of locating vaccinated subjects in rural villages to administer a second dose for routine vaccination campaigns). 

The GEO-LM01 reported here is designed to produce secreted as well as cell-associated LASV proteins in VLP (GPC+Z) formations in vaccinated subjects. This vaccine thereby provides two sources of viral antigens that mimic a natural viral infection promoting predominantly a balanced T cell response, which is critical for protection against LASV infection [30,31]. 

In our murine model, antibody responses occur too late to protect from an acute viral infection. We assessed binding antibodies in ML29-coated ELISA plates that are able to detect both the GP and Z components of VLP. Unfortunately, half of the Z protein is comprised of a RING structure that is common to many ligases and transcription factors in the normal cell, so it cross-reacts with many other proteins. In a recent publication [30], we posit that the value of the Z in VLP vectors is mostly to boost immunity by forming a 3-D structure with GP since conformational antigens are more immunogenic than linear antigens (boosting both CD4 and B cell responses). 

As a vaccine candidate, GEO-LM01 has a unique combination of advantages that could potentially meet the preferred Target Product Profiles (TPP) of a LASV vaccine set by the WHO for both non-emergency (Preventive Use) and emergency settings (Reactive/Outbreak use) [28]. These include the immunological advantages of a live but safe (replication-deficient) vector that produces conformationally relevant VLPs; inclusion of multiple genes of LASV (GPC and Z) for induction of potentially broad immunity; the ability to immunize sequentially or concurrently with other MVA-vectored vaccines without generating any vector immunity [32] (potentially even combining GEO-LM01 with other hemorrhagic fever vaccines in a multivalent formulation); the likelihood of single-dose protection, as demonstrated with MVA-VLP-EBOV and MVA-Zika vaccines candidates [32,33] with the durability of the immune response attributed to MVA-vectored vaccines; and a temperature stable, simple, adjuvant-free presentation. 

Considering the high prevalence of HIV in some LASV endemic areas of Sub-Saharan Africa (up to 68% in some populations) [34], an LASV vaccine must be safe and effective in immunocompromised populations [35]. Safety for the parental MVA vector was shown in more than 120,000 subjects in Europe, including immunocompromised individuals, during the initial development of MVA as a “safer smallpox vaccine” [36,37]. Furthermore, MVA has shown no reproductive toxicity in studies in pregnant rabbits or rats [38], and was used as a vector for malaria, influenza, and tuberculosis vaccines as well for cancer therapies in over 100 clinical trials since 1999 [32,33,39]. Our MVA-VLP HIV vaccine has demonstrated outstanding safety in clinical trials involving 500 humans [40,41], including immunocompromised individuals as well as HIV patients [40,41,42]. 

In this paper, we demonstrated that a single dose of GEO-LM01 confered full protection against the challenge virus ML29, which was directly inoculated into the brain. ML29 virus itself confers protection by the peripheral route, but its classification as a Risk group 3 (BSL3) agent creates major challenges for manufacturing in the US; in Europe it is considered Risk group 2 (BSL2). Similarly, commercial yellow fever (YF) vaccines (strains 17D and 17DD), approved more than half a century ago, are lethal for immunocompetent mice when inoculated by the IC route but can confer protection when administered by peripheral routes [43]. These strains are used as challenge viruses for the evaluation of new vaccines against YF virus. Our future work will continue in Hartley guinea pigs and NHP models (e.g., in cynomolgous macaques where signs of LASV infection mirror human disease) with live LASV under high laboratory containment, that is, Animal BSL4 (ABSL4) where the challenge virus is fully lethal by the parenteral route.

T cells are known to be a vital component of immune-mediated protection against LASV infection [44]. T cells also steer the B cell response and, therefore, a thorough investigation of the T cell response yields insight concerning the mechanisms of both cellular and humoral protection. Despite a strong T cell response, especially with CD4+ populations that are double positive for IFNγ and IL2 (an indication of expanding population), at day 11 after single-dose vaccination there was a low level of IgG antibodies produced at this timepoint. While administration of nAbs in the early stages of LASV infection by passive transfer has shown protection in animal models [45,46], the protection afforded by prior vaccination is thought to be strictly dependent on a T cell response [30,31,35,47,48]. Similarly, multiple vaccine vector platforms that showed high levels of protection in mice, guinea pigs, and NHP have not induced detectable levels of neutralizing Ab (reviewed in Ref 26). 

In the 1980s, Clegg and Lloyd [49] expressed Lassa NP from a vaccinia vector and succeeded in protecting guinea pigs, but not primates, from lethal LF disease. Since then, researchers at Porton Down have used a safer vector, MVA, to express LASV NP and protect guinea pigs from LF disease progression [50]. Since they elicited both bAb and a robust cell-mediated immunity to NP in the guinea pig model, it is possible that the MVA-LAS NP could be a standalone vaccine.

GPC is the natural target for nAb responses since it contains epitopes involved in both entry and the endosomal fusion process. GPC, however, is highly glycosylated with 11 N-linked carbohydrate sites on each monomer comprising ~25% of the total mass of the protein, shielding critical residues from Ab access. nAb against GPC have been isolated from the sera of convalescent patients, but these are rare and appear very late in infection [51]. We have consistently elicited excellent functional antibody responses (e.g., bAb, nAb and antibody-dependent cell-mediated cytotoxicity, ADCC) with MVA vaccines that target other viruses such as HIV, Ebola, and Zika [32,33,40,41,50]. A recent publication elicits excellent bAb against an MVA vector expressing LASV NP that has no glycan shield [52]. The low natural bAb against LASV GPC speaks to the effectiveness of the immuno-evasive properties of the protein, including its glycan shield, explaining failures of nAb-based vaccines or therapies using convalescent sera [51]. The combination of the 33 glycans shield, leaving only a few regions vulnerable to antibody binding, and the metastability of the native protein further explain why bAb responses are altogether difficult to elicit by vaccination. Here, we saw robust bAb at d36 (Figure 4d), but that could be attributed to the boost from ML29 virus challenge. Neutralizing antibodies have been isolated from LF survivors as patients were recovering from illness during which the humoral response had time to mature [53]. Recently, by introducing a number of mutations in GPC, this protein could be “locked” in its prefusion state increasing its stability and enabling co-crystallization with human nAb 37.7 H [16]. We are currently exploring whether replacement of the locked GPC structure with native GPC or any other modifications thereof could result in a viable vaccine candidate, demasking the GPC from carbohydrate shields and exposing more epitopes for inducing binding and neutralizing antibodies in vaccinated hosts. 

## 4. Materials and Methods 

### 4.1. Vaccine Construction, Seed Stock Preparation, VLP Formation, and Protein Expression

A recombinant GEO-LM01 was constructed on GeoVax’s MVA-VLP platform, utilizing the highly potent yet safe viral vector MVA [54,55]. The vaccine produces two LASV proteins, GPC and Z proteins from Josiah strain LASV lineage IV (GenBank JN650517.1 and JN650518.1). We used a parental MVA that had been harvested in 1974 before the appearance of Bovine Spongiform Encephalopathy /Transmissible Spongiform Encephalopathy (BSE/TSE) and sent in 2001 to Dr. Bernard Moss at NIAID, where it was plaque purified 3 times using certified reagents from sources free of BSE. The pLW76 shuttle vector (Wyatt and Moss, unpublished data) was used to insert the GPC sequences between two essential genes of MVA (I8R and G1L), and LASV Z gene into a restructured and modified deletion III between the A50R and B1R genes. These insertion sites have been identified as supporting high expression and insert stability. All inserted sequences were codon optimized for MVA. Silent mutations were introduced to interrupt homo-polymer sequences (>4G/C and >4A/T), which reduce RNA polymerase errors that possibly lead to frameshifts. The sequences were edited for vaccinia-specific terminators to remove motifs that could lead to premature termination [56]. All vaccine inserts were placed under the modified H5 early/late vaccinia promoter as described previously [57,58]. Vectors, Research Seed Virus (RSV), and Research Stocks (RS) were prepared in a dedicated room at GeoVax, with full traceability and complete documentation of all steps using BSE/TSE-free raw materials and therefore can be directly used for production of cGMP Master Seed Virus (MSV). For production of RSV for animal studies, specific pathogen-free CEF cells were acquired from Charles River Laboratories (cat#10100807), seeded into sterile tissue culture flasks, and infected with GEO-LM01 at MOIs of 0.01. Cells were recovered at 3 days post-infection, disrupted by sonication, and bulk harvest material clarified by low-speed centrifugation. The clarified viral harvest was purified using sucrose cushion ultracentrifugation. The purified viruses were titrated by limiting dilution in DF1 cells, diluted to 1 × 10^8^ TCID_50_/mL in sterile PBS + 7.5% sucrose, dispensed into sterile vials, and stored at −80 °C. Generally, a minimum of 1 × 10^10^ TCID_50_ of vaccines are produced per mL of harvest material. The VLP formation was evaluated by electron microscopy conducted on thin sections of 48 hour-infected DF-1 cell and was performed at the Emory University Apkarian Integrated Electron Microscopy Core, as previously described [59]. The expression of GPC and Z proteins by MVA was assessed by immunostaining of GEO-LM01 infected DF1 cells that had been fixed in 1:1 methanol:acetone and probed using LASV-specific GPC and Z antibodies (IBT 0307-001, rabbit anti-LASV GP pAb and IBT 0307-002, rabbit anti LASV Z pAb). Plaques were serially diluted (10-fold) from 1:100 to 1:100,000 and stained with anti-GPC or anti-Z monoclonal antibodies, respectively. Similar plaque numbers at each dilution in the GPC and Z wells show that both inserts are retained. The expression of full-length GP1 and GP2 (derived from cleavage of GPC) as well as Z protein, was confirmed by WB analysis conducted on supernatants and lysates of 293T cells 48 hours after infection using standard practices. A pool of human anti-GP1 and -GP2 antibodies (2.4F and 22.5D from Dr. James Robinson, Tulane University, New Orleans, LA) were used as the primary Abs for GP1 and GP2, and a polyclonal rabbit Ab as the primary Ab for Z (IBT Catalog# 0307-002).

### 4.2. Mouse Immunogenicity and Efficacy Studies

#### 4.2.1. **Experiment 1**. Determination of Vaccine Efficacy by Different Routes

Vaccine challenge studies were performed in a mouse model as previously described [24]. Lethality of intracerebrally (IC)-administered ML29 virus was tested in several inbred mouse models and CBA/J mice were the most sensitive to challenge. ML29 is a reassortant virus with its large genome segment from MOPV and its small segment from LASV; subcutaneous administration protects guinea pigs and primates from lethal challenge with LASV [23], but in the absence of vaccination is lethal in this IC challenge model. According to the CDC, ML29 is classified as a BSL3 agent, but not as a Select Agent. In line with University of Maryland Biosafety protocols, all propagation and experiments with high-titer stocks of ML29 virus were performed under BSL3 containment. Day-to-day experiments performed with low-titer stocks were under BSL2 containment. According to biosafety and animal care and use protocols in place, animal studies with low-titer stocks of ML29 virus were performed under ABSL2 containment. The challenge dose was selected based on uniform lethality in unprotected mice as described previously [26]. Immunization doses were based on prior studies testing efficacy of MVA vectored vaccines [38,39]. 

To determine the best route of immunization, 4–6 week-old CBA/J mice (n = 6) were immunized with 10^7^ TCID_50_ of GEO-LM01 by intraperitoneal (IP), intramuscular (IM), or subcutaneous (SC) inoculations. Two groups of mice (n = 6 each) were injected IP with ML29 at 1000 plaque-forming units (PFU) or saline, and served as positive and negative controls, respectively. On day 14, all mice were challenged by IC inoculation with 1000 PFU of ML29 and monitored for weight change, morbidity, and mortality.ML29 titers in the brains of challenged mice were not high enough to be detected by plaque assay. However, ML29 was detectable in a sensitive co-culture assay described in ref [35]. Positive (+) detection was seen in the brains of dying mice, and negative (-) in the brains of surviving mice. Since successfully vaccinated mice survived whereas those given saline or empty vectors succumbed within a week of being challenged, the MVA-Lassa vaccine proved capable of controlling ML29 replication.

#### 4.2.2. **Experiment 2**. Confirmation of Route and Immune Response

A second study to measure immunogenicity and efficacy was initiated in the same model system, in this instance examining only two conditions: immunization with GEO-LM01 by IM inoculation (n = 10) and mock immunization with saline (n = 8). Spleens were harvested from immunized animals (n = 3) and from saline-immunized animals on day 11 after a single administration of the vaccine, and antigen-specific T cell responses were measured by intracellular cytokine staining for IFNγ and IL-2 using LASV GPC immuno-dominant peptides for stimulation. The remaining animals from both groups were challenged on day 14 as in the first experiment. The serum obtained from the 3 animals sacrificed for T cell assays was additionally assayed for the presence of binding antibodies (bAb) against GPC, using ELISA.

### 4.3. Splenocyte Isolations and T Cell Assay

CBA/J mice immunized with saline or once with 10^7^ TCID_50_ GEO-LM01 vaccine were sacrificed on day 11 after immunization. Spleens were removed and homogenized through a cell strainer mounted on a 50 mL conical tube in DMEM-10 medium. Cells were centrifuged at 400× g for 5 minutes. Supernatants were aspirated and cells resuspended in 5mL 1× RBC lysis buffer. After 5 minutes at room temperature with occasional shaking, 25 mL HBSS were added to the tube, mixed by inversion, and cells centrifuged at 400× g for 5 minutes. Supernatants were discarded and pellets resuspended in 10mL DMEM-10. Isolated splenocytes were analyzed by ICS following standard protocols. In short, 10^6^ splenocytes were stimulated with 0.1µg LASV GP1 (SLYKGVYEL; amino acids 60–68) or GP2 (YLISIFLHL; amino acids 441–480) immuno-dominant epitopes identified within the GPC protein sequence [24,25,26], or mock stimulated with saline. Incubation proceeded for 6 hours at 37 °C + 5% CO_2_; GolgiPlug (BD Biosciences) was added at a concentration of 1.0 µL/mL for the last 4 hours of the incubation. The splenocytes were stained with Live/Dead Fixable Green Dead Cell Stain (ThermoFisher) at room temperature for 20 minutes. The samples were treated with Cytofix/Cytoperm (BD Biosciences) at 4 °C for 20 minutes and were stained with CD3-APC-CY7, CD4-PE-Cy7, CD8-PerCP, IL2-PE, and IFN-γ-Alexa647 (all flow cytometry antibodies from BD Biosciences). Samples were analyzed with a FACSCanto flow cytometer (BD Biosciences) utilizing FACSDiva software. Analysis was performed with FlowJo software. Responses to GP1 and GP2 stimulated cells were added together to represent the total GP stimulated response and then normalized to values from saline stimulated cells. Cytokine responses were scored as a percent of total CD8^+^ or CD4^+^ T cells.

### 4.4. ELISA

Whole blood samples were obtained by retro-orbital bleeding just prior to immunization on days 11–14 post vaccination, and at the termination of experiments. The terminal bleeds shown in Figure 4d were day 36 (22 days following challenge). Sera were obtained by centrifugation of the whole blood samples in serum separator tubes at 3500× g for 5 min. and used to measure bAb in ELISA as described previously [60]. Briefly, flat-bottom 96-well plates were coated overnight at 4 °C with sonication-inactivated ML29 virus, 10^5^ PFU/mL in 100 mM bicarbonate buffer, pH 9.6. Plates were washed 4 times with phosphate buffered saline (PBS) + 0.05% Tween-20 (PBST). Next, the plates were blocked with StartingBlock Blocking Buffer (Thermo Fisher Scientific) for 5 minutes at room temperature. Serially-diluted heat-inactivated mouse serum samples were added to wells and incubated for 1 hour at 37 °C. After washing with PBST, goat anti-mouse IgG-HRP (ImmunoReagents) was added to each well and incubated for 1 hour at 37 °C. After a final washing, SureBlue TMB (3,3′, 5, 5′—Tetramethylbenzidine) 1-component substrate solution (KPL) was added to each well, incubated in the dark at room temperature for 10 minutes, and stopped with 1N hydrochloric acid (HCl). Optical density at 450 nm was determined using a Vmax Kinetic ELISA Absorbance Microplate Reader (Molecular Devices). Normalized absorbance values were determined by first subtracting optical density values of blank negative control wells. Endpoint titer was determined by the dilution at which the optical density equaled 0.03. 

### 4.5. Statistical Analyses 

Analysis of data was performed using GraphPad Prism (GraphPad Software). Comparisons between groups were performed using a Student’s t-test. To test for pairwise differences between groups, an analysis of variance (ANOVA) and Tukey’s test of multiple comparisons was performed. Correlations were performed by Spearman rank-correlation tests. A *p*-value less than 0.05 was considered statistically significant.

### 4.6. Regulatory Compliance 

All methods were carried out in accordance with the relevant guidelines and regulations. All experimental protocols were approved by the University of Maryland School of Medicine Institutional Animal Care and Use Committee (IACUC Protocol # 0217017) and the Institutional Biosafety Committee (approval # IBC-00001495).

## 5. Conclusions

GEO-LM01 uses GeoVax’s MVA-VLP vaccine platform that has been shown to be safe and to induce durable antibody and T cell responses in multiple human clinical trials for GeoVax’s prophylactic HIV vaccine. In contrast to other LF vaccines in development that rely on a single glycoprotein antigen, GEO-LM01 includes an additional matrix protein that forms VLPs with the LASV glycoproteins. Upon infection of human cells, the MVA-driven expression of the two proteins leads to the formation of non-infectious VLPs that bear natively-folded glycoproteins on their surface, triggering an effective immune response that is predicted to be more broadly cross-protective than vaccines expressing only one viral antigen [30]. We previously demonstrated that our MVA-VLP-Ebola vaccine conferred full protection after a single dose in non-human primates. This vaccine induced near sterile immunity since no live virus could be detected in the blood of any vaccinated animal post challenge compared to controls (>5log_10_ TCID_50_ per mL of blood). We are currently evaluating the efficacy of our LF vaccine in guinea pigs and non-human primate models. It remains to be seen whether this vaccine will also be a best-in class vaccine for LF, as shown for the MVA-VLP-Ebola vaccine [61]. 

## Figures and Tables

**Table 1:** Surviving mice from Experiment 2 were re-challenged ^1^ one year later.

Experimental Mice	Number of Mice	Survivors after 2nd Challenge
Survivors of 1st challenge	4	4
Control mice (naïve adult CBA/J)	4	0

^1^ All mice were challenged with a lethal dose of ML29 to see if the original vaccinated survivors could survive a second lethal challenge a year later. The naïve mice served as negative controls.

## References

[1] WHO Epidemic Focus: Lassa fever Weekly Epidemiological Record. https://www.who.int/wer/2016/wer9121.pdf?ua=1.

[2] Shao J., Liang Y., Ly H. (2015). Human Hemorrhagic Fever Causing Arenaviruses: Molecular Mechanisms Contributing to Virus Virulence and Disease Pathogenesis. Pathogens.

[3] Leski T.A., Stockelman M.G., Moses L.M., Park M., Stenger D.A., Ansumana R., Bausch D.G., Lin B. (2015). Sequence Variability and Geographic Distribution of Lassa Virus, Sierra Leone. Emerg. Infect. Dis..

[4] McCormick J.B., Webb P.A., Krebs J.W., Johnson K.M., Smith E.S. (1987). A Prospective Study of the Epidemiology and Ecology of Lassa Fever. J. Infect. Dis..

[5] McCormick J.B., Saluzzo J.F., Dodet B. (1999). Lassa Fever, in Emergence and Control of Rodent-Borne Viral Diseases.

[6] Richmond J.K., Baglole D.J. (2003). Lassa fever: Epidemiology, clinical features, and social consequences. BMJ.

[7] Control N.C.f.D. (2018). Disease Situation Reports. https://ncdc.gov.ng/diseases/sitreps.

[8] Olayemi A., Cadar D., Magassouba N.F., Obadare A., Kourouma F., Oyeyiola A., Fasogbon S., Igbokwe J., Rieger T., Bockholt S. (2016). New Hosts of The Lassa Virus. Sci. Rep..

[9] Fisher-Hoch S.P., Tomori O., Nasidi A., I Perez-Oronoz G., Fakile Y., Hutwagner L., McCormick J.B. (1995). Review of cases of nosocomial Lassa fever in Nigeria: The high price of poor medical practice. BMJ.

[10] Mylne A.Q.N., Pigott D.M., Longbottom J., Shearer F., Duda K.A., Messina J.P., Weiss D.J., Moyes C.L., Golding N., Hay S.I. (2015). Mapping the zoonotic niche of Lassa fever in Africa. Trans. R. Soc. Trop. Med. Hyg..

[11] Bowen M.D., Rollin P.E., Ksiazek T.G., Hustad H.L., Bausch D.G., Demby A.H., Bajani M.D., Peters C.J., Nichol S.T. (2000). Genetic Diversity among Lassa Virus Strains. J. Virol..

[12] Hallam H.J., Hallam S., Rodriguez S.E., Barrett A.D.T., Beasley D.W.C., Chua A., Ksiazek T.G., Milligan G.N., Sathiyamoorthy V., Reece L.M. (2018). Baseline mapping of Lassa fever virology, epidemiology and vaccine research and development. NPJ Vaccines.

[13] Radoshitzky S.R., Bao Y., Buchmeier M.J., Charrel R.N., Clawson A.N., Clegg C.S., DeRisi J.L., Emonet S., Gonzalez J.-P., Kuhn J.H. (2015). Past, present, and future of arenavirus taxonomy. Arch. Virol..

[14] Djavani M., Lukashevich I.S., Sanchez A., Nichol S.T., Salvato M.S. (1997). Completion of the Lassa fever virus sequence and identification of a RING finger ORF at the L RNA 5′ end. Virology.

[15] Lukashevich I.S., Nichol S.T., Shapiro K., Ravkov E., Sanchez A., Djavani M., Salvato M.S. (1997). The Lassa fever virus L gene: Nucleotide sequence, comparison, and precipitation of a predicted 250 kDa protein with monospecific antiserum. J. Gen. Virol..

[16] Hastie K.M., Zandonatti M.A., Kleinfelter L.M., Heinrich M.L., Rowland M.M., Chandran K., Branco L.M., Robinson J.E., Garry R.F., Saphire E.O. (2017). Structural basis for antibody-mediated neutralization of Lassa virus. Science.

[17] Lenz O., Ter Meulen J., Klenk H.-D., Seidah N.G., Garten W. (2001). The Lassa virus glycoprotein precursor GP-C is proteolytically processed by subtilase SKI-1/S1P. Proc. Natl. Acad. Sci. USA.

[18] Ölschläger S., Flatz L. (2013). Vaccination Strategies against Highly Pathogenic Arenaviruses: The Next Steps toward Clinical Trials. PLoS Pathog..

[19] Urata S., Yasuda J. (2015). Cis- and cell-type-dependent trans-requirements for Lassa virus-like particle production. J. Gen. Virol..

[20] Safronetz D., Mire C., Rosenke K., Feldmann F., Haddock E., Geisbert T., Feldmann H. (2015). A Recombinant Vesicular Stomatitis Virus-Based Lassa Fever Vaccine Protects Guinea Pigs and Macaques against Challenge with Geographically and Genetically Distinct Lassa Viruses. PLoS Negl. Trop. Dis..

[21] Verheust C., Goossens M., Pauwels K., Breyer D. (2012). Biosafety aspects of modified vaccinia virus Ankara (MVA)-based vectors used for gene therapy or vaccination. Vaccine.

[22] Hastie K.M., Zandonatti M., Liu T., Li S., Woods V.L., Saphire E.O. (2016). Crystal Structure of the Oligomeric Form of Lassa Virus Matrix Protein Z. J. Virol..

[23] Lukashevich I.S., Patterson J., Carrion R., Moshkoff D., Ticer A., Zapata J., Brasky K., Geiger R., Hubbard G.B., Bryant J. (2005). A Live Attenuated Vaccine for Lassa Fever Made by Reassortment of Lassa and Mopeia Viruses. J. Virol..

[24] Goicochea M.A., Zapata J.C., Bryant J., Davis H., Salvato M.S., Lukashevich I.S. (2012). Evaluation of Lassa virus vaccine immunogenicity in a CBA/JML29 mouse model. Vaccine.

[25] Botten J., Alexander J., Pasquetto V., Sidney J., Barrowman P., Ting J., Peters B., Southwood S., Stewart B., Rodriguez-Carreno M.P. (2006). Identification of Protective Lassa Virus Epitopes That Are Restricted by HLA-A2. J. Virol..

[26] Boesen A., Sundar K., Coico R. (2005). Lassa Fever Virus Peptides Predicted by Computational Analysis Induce Epitope-Specific Cytotoxic-T-Lymphocyte Responses in HLA-A2.1 Transgenic Mice. Clin. Vaccine Immunol..

[27] Kyei N.N., Abilba M.M., Kwawu F.K., Agbenohevi P.G., Bonney J.H., Agbemaple T.K., Nimo-Paintsil S.C., Ampofo W., Ohene S.-A., Nyarko E.O. (2015). Imported Lassa fever: A report of 2 cases in Ghana. BMC Infect. Dis..

[28] Mehand M.S., Al Shorbaji F., Millett P., Murgue B. (2018). The WHO R&D Blueprint: 2018 review of emerging infectious diseases requiring urgent research and development efforts. Antivir. Res..

[29] CEPI (2018). News. http://cepi.net/news.

[30] Zapata J.C., Medina-Moreno S., Guzmán-Cardozo C., Salvato M.S. (2018). Improving the Breadth of the Host’s Immune Response to Lassa Virus. Pathogens.

[31] Ter Meulen V. (1999). Lassa fever: Implications of T-cell immunity for vaccine development. J. Biotechnol..

[32] Domi A., Feldmann F., Basu R., McCurley N., Shifflett K., Emanuel J., Hellerstein M.S., Guirakhoo F., Orlandi C., Flinko R. (2018). A Single Dose of Modified Vaccinia Ankara expressing Ebola Virus Like Particles Protects Nonhuman Primates from Lethal Ebola Virus Challenge. Sci. Rep..

[33] Brault A.C., Domi A., McDonald E.M., Talmi-Frank D., McCurley N., Basu R., Robinson H.L., Hellerstein M., Duggal N.K., Bowen R.A. (2017). A Zika Vaccine Targeting NS1 Protein Protects Immunocompetent Adult Mice in a Lethal Challenge Model. Sci. Rep..

[34] Djomand G., Quaye S., Sullivan P.S. (2014). HIV epidemic among key populations in west Africa. Curr. Opin. HIV AIDS.

[35] Zapata J.C., Poonia B., Bryant J., Davis H., Ateh E., George L., Crasta O., Zhang Y., Slezak T., Jaing C. (2013). An attenuated Lassa vaccine in SIV-infected rhesus macaques does not persist or cause arenavirus disease but does elicit Lassa virus-specific immunity. Virol. J..

[36] Sutter G., Moss B. (1992). Nonreplicating vaccinia vector efficiently expresses recombinant genes. Proc. Natl. Acad. Sci. USA.

[37] Overton E.T., Stapleton J., Frank I., Hassler S., Goepfert P.A., Barker D., Wagner E., Von Krempelhuber A., Virgin G., Weigl J. (2015). Safety and Immunogenicity of Modified Vaccinia Ankara-Bavarian Nordic Smallpox Vaccine in Vaccinia-Naive and Experienced Human Immunodeficiency Virus-Infected Individuals: An Open-Label, Controlled Clinical Phase II Trial. Open Forum Infect. Dis..

[38] Committee for Medicinal Products for Human Use (CHMP), Committee for Medicinal Products for Human Use (CHMP) (2013). Assessment Report, IMVANEX, Common Name: Modified Vaccinia Ankara Virus, Procedure No. EMEA/H/C/002596.

[39] Bliss C.M., Bowyer G., Anagnostou N.A., Havelock T., Snudden C.M., Davies H., De Cassan S.C., Grobbelaar A., Lawrie A.M., Venkatraman N. (2018). Assessment of novel vaccination regimens using viral vectored liver stage malaria vaccines encoding ME-TRAP. Sci. Rep..

[40] Goepfert P.A., Elizaga M.L., Sato A., Qin L., Cardinali M., Hay C.M., Hural J., DeRosa S.C., Defawe O.D., Tomaras G.D. (2011). Phase 1 Safety and Immunogenicity Testing of DNA and Recombinant Modified Vaccinia Ankara Vaccines Expressing HIV-1 Virus-like Particles. J. Infect. Dis..

[41] Goepfert P.A., Elizaga M.L., Seaton K., Tomaras G.D., Montefiori D.C., Sato A., Hural J., DeRosa S.C., Kalams S.A., McElrath M.J. (2014). Specificity and 6-Month Durability of Immune Responses Induced by DNA and Recombinant Modified Vaccinia Ankara Vaccines Expressing HIV-1 Virus-Like Particles. J. Infect. Dis..

[42] Thompson M., Heath S.L., Sweeton B., Williams K., Cunningham P., Keele B.F., Sen S., Palmer B.E., Chomont N., Xu Y. (2016). DNA/MVA Vaccination of HIV-1 Infected Participants with Viral Suppression on Antiretroviral Therapy, followed by Treatment Interruption: Elicitation of Immune Responses without Control of Re-Emergent Virus. PLoS ONE.

[43] Maciel M.M., Cruz F.D.S.P., Cordeiro M.T., da Motta M.A., de Melo Cassemiro K.M.S., Maia R.D.C.C., de Figueiredo R.C.B.Q., Galler R., da Silva Freire M., August J.T. (2015). A DNA Vaccine against Yellow Fever Virus: Development and Evaluation. PLoS Negl. Trop. Dis..

[44] Perdomo-Celis F., Salvato M.S., Medina-Moreno S., Zapata J.C. (2019). T-Cell Response to Viral Hemorrhagic Fevers. Vaccines.

[45] Cross R.W., Mire C.E., Branco L.M., Geisbert J.B., Rowland M.M., Heinrich M.L., Goba A., Momoh M., Grant D.S., Fullah M. (2016). Treatment of Lassa virus infection in outbred guinea pigs with first-in-class human monoclonal antibodies. Antivir. Res..

[46] Mire C.E., Cross R.W., Geisbert J.B., Borisevich V., Agans K.N., Deer D.J., Heinrich M.L., Rowland M.M., Goba A., Momoh M. (2017). Human-monoclonal-antibody therapy protects nonhuman primates against advanced Lassa fever. Nat. Med..

[47] Lukashevich I.S., Carrion R., Salvato M.S., Mansfield K., Brasky K., Zapata J., Cairo C., Goicochea M., Hoosien G.E., Ticer A. (2008). Safety, immunogenicity, and efficacy of the ML29 reassortant vaccine for Lassa fever in small non-human primates. Vaccine.

[48] Kiley M. (1979). Protection of Rhesus Monkeys from Lassa Virus by Immunisation With Closely Related Arenavirus. Lancet.

[49] Clegg J., Lloyd G. (1987). Vaccinia Recombinant Expressing Lassa-Virus Internal Nucleocapsid Protein Protects Guinea pigs against Lassa Fever. Lancet.

[50] McCurley N.P., Domi A., Basu R., Saunders K.O., Labranche C.C., Montefiori D.C., Haynes B.F., Robinson H.L. (2017). HIV transmitted/founder vaccines elicit autologous tier 2 neutralizing antibodies for the CD4 binding site. PLoS ONE.

[51] Sommerstein R., Flatz L., Remy M.M., Malinge P., Magistrelli G., Fischer N., Sahin M., Bergthaler A., Igonet S., Ter Meulen J. (2015). Arenavirus Glycan Shield Promotes Neutralizing Antibody Evasion and Protracted Infection. PLoS Pathog..

[52] Kennedy E., Dowall S., Salguero F., Yeates P., Aram M., Hewson R. (2019). A vaccine based on recombinant modified Vaccinia Ankara containing the nucleoprotein from Lassa virus protects against disease progression in a guinea pig model. Vaccine.

[53] Robinson J.E., Hastie K.M., Cross R.W., Yenni R.E., Elliott D.H., Rouelle J.A., Kannadka C.B., Smira A.A., Garry C.E., Bradley B.T. (2016). Most neutralizing human monoclonal antibodies target novel epitopes requiring both Lassa virus glycoprotein subunits. Nat. Commun..

[54] Altenburg A.F., Kreijtz J.H.C.M., De Vries R.D., Song F., Fux R., Rimmelzwaan G.F., Sutter G., Volz A. (2014). Modified Vaccinia Virus Ankara (MVA) as Production Platform for Vaccines against Influenza and Other Viral Respiratory Diseases. Viruses.

[55] Moss B., Carroll M.W., Wyatt L.S., Bennink J.R., Hirsch V.M., Goldstein S., Elkins W.R., Fuerst T.R., Lifson J.D., Piatak M. (1996). Host Range Restricted, Non-Replicating Vaccinia Virus Vectors as Vaccine Candidates. Results Probl. Cell Differ..

[56] Wyatt L.S., Earl P.L., Vogt J., Eller L.A., Chandran D., Liu J., Robinson H.L., Moss B. (2008). Correlation of immunogenicities and in vitro expression levels of recombinant modified vaccinia virus Ankara HIV vaccines. Vaccine.

[57] Wyatt L.S., Earl P.L., Xiao W., Americo J.L., Cotter C.A., Vogt J., Moss B. (2009). Elucidating and Minimizing the Loss by Recombinant Vaccinia Virus of Human Immunodeficiency Virus Gene Expression Resulting from Spontaneous Mutations and Positive Selection. J. Virol..

[58] Earl P.L., Cotter C., Moss B., VanCott T., Currier J., Eller L.A., McCutchan F., Birx D.L., Michael N.L., Marovich M.A. (2009). Design and evaluation of multi-gene, multi-clade HIV-1 MVA vaccines. Vaccine.

[59] Hellerstein M., Xu Y., Marino T., Lu S., Yi H., Wright E.R., Robinson H.L. (2012). Co-expression of HIV-1 virus-like particles and granulocyte-macrophage colony stimulating factor by GEO-D03 DNA vaccine. Hum. Vaccines Immunother..

[60] Salvato M.S., Lukashevich I.S., Medina-Moreno S., Zapata J.C. (2018). Diagnostics for Lassa Fever: Detecting Host Antibody Responses, in Hemorrhagic Fever Viruses: Methods and Protocols.

[61] Lázaro-Frías A., Gomez-Medina S., Sánchez-Sampedro L., Ljungberg K., Ustav M., Liljeström P., Muñoz-Fontela C., Esteban M., García-Arriaza J. (2018). Distinct Immunogenicity and Efficacy of Poxvirus-Based Vaccine Candidates against Ebola Virus Expressing GP and VP40 Proteins. J. Virol..

